# μ Opioid Receptor Expression after Morphine Administration Is Regulated by miR-212/132 Cluster

**DOI:** 10.1371/journal.pone.0157806

**Published:** 2016-07-05

**Authors:** Adrian Garcia-Concejo, Ada Jimenez-Gonzalez, Raquel E. Rodríguez

**Affiliations:** 1 Institute of Neurosciences of Castilla y Leon (INCyL), C/Pintor Fernando Gallego, 1, 37007, Salamanca, Spain; 2 Department of Biochemistry and Molecular Biology, Faculty of Medicine, University of Salamanca, C/ Alfonso X El Sabio, 0 S-N Campus Miguel De Unamuno, 37007, Salamanca, Spain; 3 Institute of Biomedical Research of Salamanca (IBSAL), Hospital Universitario de Salamanca—Edificio Virgen de la Vega. Décima Planta, P° de San Vicente 58–182, 37007, Salamanca, Spain; Xi'an Jiaotong University School of Medicine, CHINA

## Abstract

Since their discovery, miRNAs have emerged as a promising therapeutical approach in the treatment of several diseases, as demonstrated by miR-212 and its relation to addiction. Here we prove that the miR-212/132 cluster can be regulated by morphine, through the activation of mu opioid receptor (Oprm1). The molecular pathways triggered after morphine administration also induce changes in the levels of expression of *oprm1*. In addition, miR-212/132 cluster is actively repressing the expression of mu opioid receptor by targeting a sequence in the 3’ UTR of its mRNA. These findings suggest that this cluster is closely related to opioid signaling, and function as a post-transcriptional regulator, modulating morphine response in a dose dependent manner. The regulation of miR-212/132 cluster expression is mediated by MAP kinase pathway, CaMKII-CaMKIV and PKA, through the phosphorylation of CREB. Moreover, the regulation of both *oprm1* and of the cluster promoter is mediated by MeCP2, acting as a transcriptional repressor on methylated DNA after prolonged morphine administration. This mechanism explains the molecular signaling triggered by morphine as well as the regulation of the expression of the mu opioid receptor mediated by morphine and the implication of miR-212/132 in these processes.

## Introduction

The analysis of the molecular signalling cascade activated by morphine may lead to unveil new molecular targets that could be used to palliate the undesired effects of opiate drugs. The opioid receptors have been widely studied, and their regulation after being activated could explain the molecular basis of addiction, especially the mu opioid receptor (Oprm1), since it is the receptor which morphine binds to with the highest affinity [1]. Opioid receptors are G-protein coupled receptors, and the signalling cascade they activate induces the activation of cAMP Response Element Binding (CREB) protein [2, 3], a transcription factor involved in many molecular processes.

Since their discovery, microRNAs (miRNAs) have emerged as extremely promising tools in the modulation of the expression of many genes [4]. miRNAs are small non coding RNA transcripts that regulate gene expression at the post-transcriptional level. These molecules control gene expression by binding to complementary sequences in the 3′ untranslated region (3′ UTR) of their target mRNA transcripts to facilitate their degradation and/or inhibit their translation [5], although binding to other regions have been described [6, 7]. miRNAs are involved in many developmental processes, such as differentiation, proliferation, and their dysregulation has been linked to a wide variety of diseases [8, 9]. Several other miRNAs, such as miR-let7d and miR-133b [10, 11], have been described to be regulated by morphine and play a role in behavioural effects of this drug.

miR-212 and miR-132 form a cluster, although their functions may not be fully correlated [12, 13]. Previous studies [14, 15] reported that expression of miR-212 was increased in the dorsal striatum of rats with extended but not restricted daily access to intravenous cocaine self-administration. Moreover, CREB activation has been shown to regulate miR-212 expression after exposure to cocaine in murine models [16]. Taking these results into consideration, it is of interest to unravel whether the regulation mechanism triggered by cocaine could also be activated by morphine through the activation of Oprm1, or more importantly, if both drugs share the functional cascade that describe their activity. The results obtained in this work show that morphine is regulating the expression of miR-212 through CREB signalling, and that miR-212 can also modulate miR-132 and *oprm1* expression inhibiting its translation by binding to its 3’ UTR. This regulation mechanism could explain some of the molecular components that remain unclear concerning opioid signalling.

## Material and Methods

### Experimental animals

Zebrafish from the AB strain were bred and raised in the Fish Facilities of our lab following standard protocols [17]. In all experiments, adequate measures were taken to minimize pain or discomfort and animals were handled according to the guidelines of the European Communities Council Directive 2010/63/UE, to the current Spanish legislation for the use and care of animals RD 53/2013 and to the Guide for the Care and Use of Laboratory animals as adopted and promulgated by the U.S. National Institutes of Health. For ISH experiments, embryos were fixed in 4% paraformaldehyde, and for RNA extraction embryos were frozen in liquid nitrogen. These experiments were approved by the University of Salamanca Ethics Committee.

### Drug Treatment and inhibitors

Zebrafish embryos were exposed to 10 nM or 10μM morphine-sulphate in E3 embryo buffer from 5 hours post fertilization (hpf) (50% epiboly) to 24/48 hpf. Fresh morphine solution was added at 24 hpf. A control group (E3 only) was used in parallel for each experiment. Morphine was provided by the Spanish Ministry of Health. Concentrations used for each inhibitor were: U0126 (Promega) 20μM, N-(6-Aminohexyl)-5-chloro-1-naphthalenesulfonamide hydrochloride (W7, Sigma) 10μM and Forskolin (Sigma) 20μM.

### RNA Extraction and qPCR

Total RNA, including miRNA, was extracted using Trizol (Life Technologies), following the manufacturers protocol. To study the levels of expression of the miRNAs of interest, a previous polyadenylation was performed using Poli-A polymerase (New England Biolabs) and a retrotranscription reaction using M-MuLV retrotranscriptase (New England Biolabs), as described in the manufacturer’s protocol. The retrotranscription of *oprm1* samples were carried out using the High Capacity RT Kit (Applied Biosystems). The absolute quantification of the PCR products was accomplished with a standard curve using the SYBR-Green method, and specifically designed oligonucleotides as described by Balcells et al., [18] The SYBR-Green was included in a 2× Master Mix (Life Technologies, SYBR Green PCR Master Mix). The final volume of each reaction was 20 μl, distributed as follows: 10 μl of Master Mix, 0.8 μl of each oligonucleotide, 7.4 μl of distilled water, and 1 μl of cDNA in a concentration of 25 ng/μl. A standard curve was constructed for each experiment by serial dilutions of cDNA. The amplification reaction were performed in an ABI 7300 qPCR Termal Cycler (Applied Biosystems), with the following conditions: 15 min at 95°C followed by 35 cycles of 15 s at 95°C, 30 s at 57°C, and 30 s at 70°C. qPCR was performed by triplicate, and each experiment was repeated with three different samples. The sequences of the primers used were the following: oprm1 qF, ACGAGCTGTGCAAGATTGTG; oprm1 qR, CCGATTGCAGATGAAAGAT; dre-miR-132 qF, AGTAAACAGTCTACAGCCATG; dre-miR-132 qR, TCCATTTTTTTTTTTTTTTTTGTCG; dre-miR-212 qF, CGCAGTAACAGTCTACAGTC; dre-miR-212 qR, CCAGTTTTTTTTTTTTTTTTTTGCCAT; pri-miR-F, TCTGGCAACCTTCCACAG, pri-miR-R, TGGAGACAGCAGGAGGG.

### Total protein extraction, immunoprecipitation and western blot

Total proteins were extracted from zebrafish embryos at 24 and 48 hpf using cold Ringer solution and 300 μl of protein extraction buffer (10 mM tris pH 7.4, 2% triton X 100, 1 mM PMSF, 1 μl/ml protease inhibitors [Sigma]). Embryos were aspired using a syringe and centrifuged 10 minutes at 10000 g at 4°C. The supernatant containing proteins was frozen at -80°C. Proteins were quantified by Bradford method. 350 μg of total protein were used for the immunoprecipitation of each group and incubated with 25 μl of Oprm1 antibody (Spanish patent license: P201330193) conjugated with protein A agarose beads. Beads were washed with lysis buffer (50 mM Tris-HCl pH 7.5, 10 mM EDTA, 1% SDS, 1x protease inhibitors) and centrifuged at 14000 rpm. Pellets were resuspended in 25 μl of 4X loading buffer (10% SDS, 600 μl of glycerol, 150 μl of 2-mercaptoethanol, 1M Tris-HCl pH: 6.8 and 30 μl of Bromophenol blue). Samples were boiled for 5 minutes and centrifuged at 14000 rpm. Supernatants were loaded in 10% polyacrylamide SDS gels. After electrophoresis, proteins were transferred to a nitrocellulose membrane and blocked with 5% milk in TBS. Oprm1 primary antibody was used at 0.25 μg/ml to detect the immunoprecipitated protein. After incubation, membranes were washed with TBS containing 20% Tween 20 and incubated for 1 hour with the goat anti-rabbit IgG-HRP secondary antibody (Santa Cruz Biotechnology) diluted 1:5000. Blots were developed with ECL (Amersham). Experiments were performed in triplicates and quantified with ImageJ software.

### Morpholino microinjection

The morpholino antisense (MO) oligomers used to knock down *oprm1*, *miR-212* and *miR-132* were purchased from Gene Tools, LLC. The MO was diluted in sterilized water to a stock concentration of 1 mM. In addition to the three MO experimental groups (untreated, 10 nM morphine, and 10 μM morphine), each experiment included a control MO group injected with morpholino that exhibits no binding target or biological activity (control morpholino). Morpholino was injected into the yolk at the one cell stage according to the published protocols [19]. The concentrations of *target* MO and control MO used were 0.33 and 1 μM, respectively (3 nl were injected into each embryo). The sequences of the morpholinos used were the following: oprm1 MO, AATGTTGCCAGTGTTTTCCATCATG; dre-miR-132 MO, ACAGTAACAATCTAATGCCACGGTC; dre-miR-212 MO, AGCCATGACTGTAGACTGTTACTGT.

### Whole-mount in Situ Hybridization (WISH)

Embryos at 24 hpf and 48 hpf were dechorionated, fixed with 4% paraformaldehyde (PFA) in phosphate saline buffer (PBS) overnight at 4°C, washed twice in PBS for 5 min at room temperature (RT), and stored in absolute methanol at −20°C until use. The hybridization was carried out as described in Sanchez-Simon et al. [20], and the probes used for this experiment were designed as described by He et al. [21].

### Luciferase assay

For chemiluminescence detection we used the Dual-Glo Luciferase Assay (Promega). The sequences were cloned into PmirGlo (Promega) using the protocol for digestion, ligation and transformation described in GeneClip™ U1 Hairpin Cloning Systems (Promega). HEK293 cells were transfected with a wild-type and mutated construct using Lipofectamine 2000 [22]. Briefly, 300ng of reporter plasmid were diluted in 25 μl of DMEM in combination with 20 nM or 50nM of miR-212 or miR-132 miRNAs mimics (small, chemically modified double-stranded RNAs that mimic endogenous miRNAs by up-regulation of miRNA activity) or 50nM of miR-1 mimics (Ambion) when required. This solution was combined with diluted Lipofectamine (1,5 μl/well) and the transfection was carried out for 48 hours at 37°C in a 5% CO_2_ incubator. Luminescence was measured during 10 seconds as recommended by the manufacturer using a luminometer (Appliskan, Thermo Scientific). The ratio between luminescence from firefly and the control (*Renilla)* was used to check if the miRNA was binding to the sequence cloned into the plasmid. The mean for each group (n = 8) was calculated and normalized with the wells transfected only with the empty plasmid. The primers cloned into the vector were the following: wtF, CTAGCTAGGTACCTAGTTAGTTTGAGGAAAGACTGTTGG; wtR, TCGACCAACAGTCTTTCCTCAAACTAACTAGGTACCTAG; misF, CTAGCTAGGTACCTAGTTAGTTTGAGGAAATCAGTGTGG; misR, TCGACCACACTGATTTCCTCAAACTAACTAGGTACCTAG

### Primary neuron culture

Brains from 42 hpf zebrafish embryos were placed in 1x E2 medium (15 mM NaCl, 0.5 mM KCl, 1 mM MgSO_4_, 150 μM KH_2_PO_4_, 50μM Na_2_HPO_4_, 1 mM CaCl_2_, 0.7 mM NaHCO_3_, 0.5 mg/l methylene blue) with 1x tricaine as described by Chen et al. [23]. Briefly, cells were grown in Dulbecco’s modified medium to 48 hpf. Morphine (1 nM) was added after plating. 48 hours later neurons were fixed in 4% paraformaldehyde (PFA) (w/v). Immunocytochemistry analysis for primary cultured cells was carried out as described by Chen et al. [23]. Neurons were fixed with 4% PFA (w/v) for 20 minutes and blocked with antibody buffer and 5% goat serum; 1:250 dilution was used for Oprm1 antibody. To-PRO (1:1000, ThermoFisher) and Alexa fluor 594 Phalloidin (1:10, ThermoFisher) were used as nuclei and soma markers, respectively. Primary cultures were analyzed with a life cell microscopy (Zeiss).

### Statistical analysis

qPCR ct values were first included in REST-384 v2 software to calculate the relative expression of groups respect to control embryos. Data were analysed by one-way analysis of variance (one-way ANOVA). Tukey’s post hoc test was then performed for multiple comparisons. Differences were considered significant at p < 0.05. Results are shown as means ± S.E.M. All statistical analyses were performed with Prism 5 (GraphPad Software, Inc).

## Results

### miR-212 and miR-132 temporal expression is closely related

**Fig 1:**
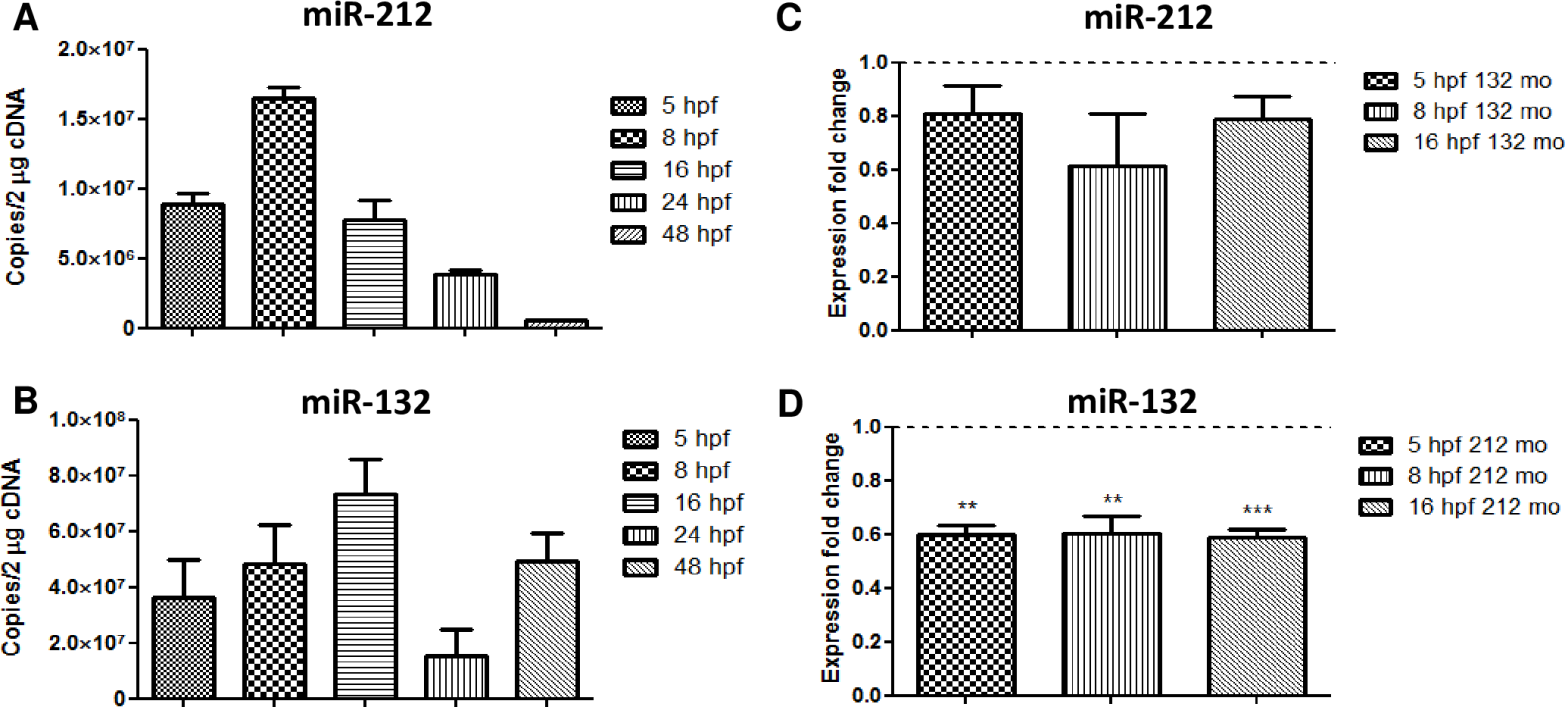
**miR-212 (A) and miR-132 (B) temporal expression, and miR-212 expression levels in 132 morphant embryos (C), and miR-132 expression levels in 212 morphant embryos (D). Mo: morphant.** The results show differences in the temporal expression of the clustered microRNAs. Figs C and D show a downregulation of one of the miRNAs when the other is knocked-down, indicating a possible regulation between the expression of these molecules in the context of their cluster. Results are shown as the number of copies per 2μg of cDNA (A and B) or the fold change respect to control levels at 24 and 48 hpf. (C and D) Data are expressed as mean ± SEM. Control group is shown as the dotted line. Statistical significance; *, p < 0,05; **, p < 0,01; ***, p < 0,001.

miR-212 and miR-132 form a genetic cluster [12, 13], and its expression and function may be related to each other. To determine the relevance of miR-212 and miR-132 during development, we studied their temporal expression at 5, 8, 16, 24 and 48 hpf (Fig 1A and 1B). Although both are highly expressed between 5 and 16 hpf, miR-212 expression decays during development. In addition, we have observed an increase in expression of miR-212 at 8 hpf and an increase of miR-132 at 16 hpf. Besides, the number of copies for miR-132 is greatly increased at 48 hpf when compared to miR-212 (Fig 1B), suggesting the importance of this miRNA at this particular stage. To study a possible relation of the levels of miRNAs, we knocked-down miR-212 and miR-132 in order to analyze the independence of each other of their expression profiles (Fig 1C and 1D). After the knock-down of miR-212, and at several stages (5, 8 and 16 hpf), we have observed a decrease in expression of miR-132 (Fig 1C). In addition, the levels of miR-212 were downregulated in miR-132 morphants (Fig 1D). Even though miR-132 is more expressed during development, miR-212 has been linked to addiction [16] and it is probably crucial during the first stages of development.

### Morphine modifies the levels of expression of miR-212, of pri-miR-212/132 and *oprm1*

**Fig 2:**
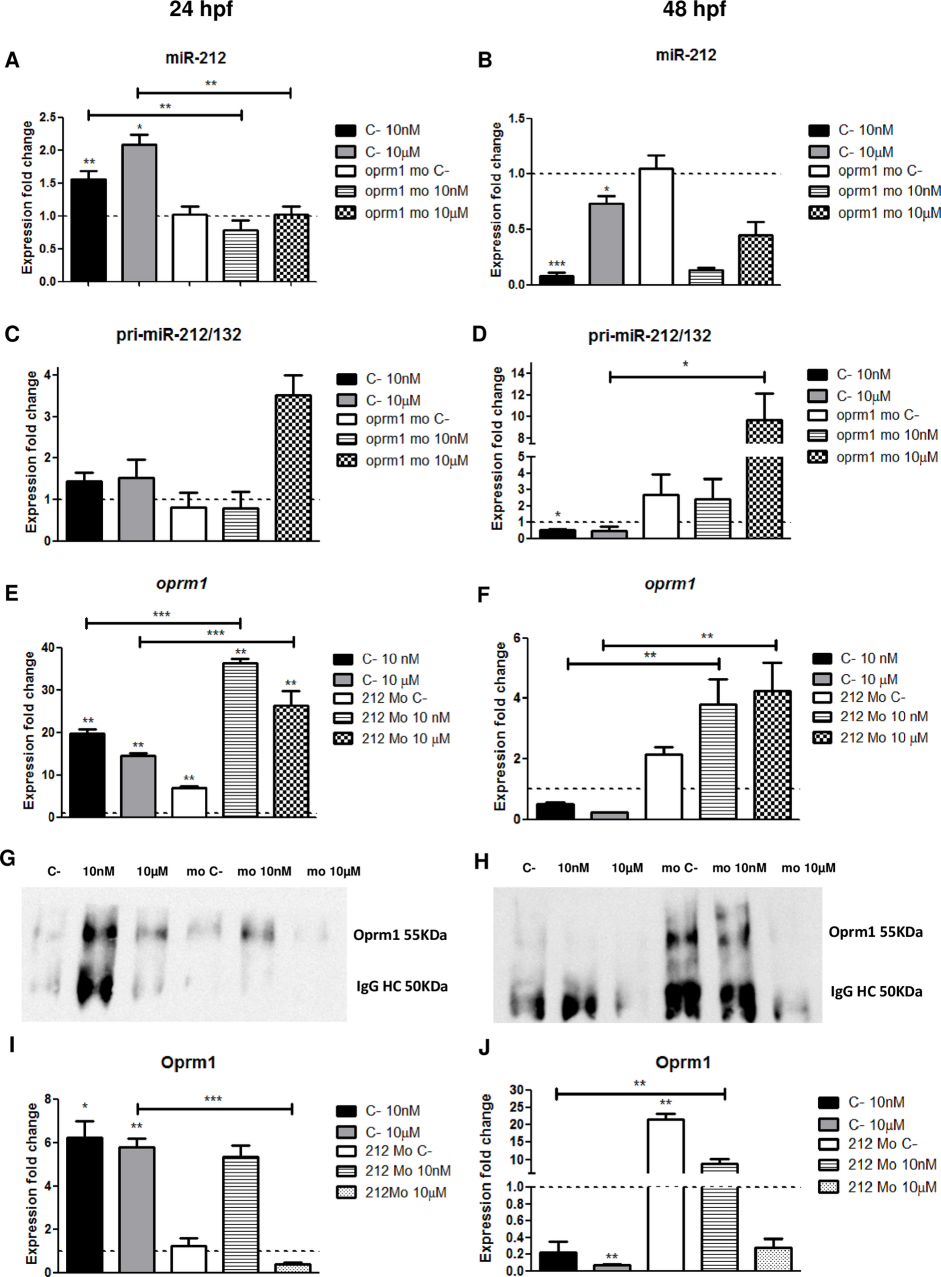
**miR-212 expression levels in control and *oprm1* morphant embryos after morphine (10 nM and 10μM) treatment (A, 24hfp; B, 48hpf). pri-miR-212/132 expression levels in control and *oprm1* morphant embryos after morphine administration (10 nM and 10μM) (C, 24hfp; D, 48hpf). *oprm1* expression levels in control and miR-212 morphant embryos after morphine (10 nM and 10μM) treatment (E, 24hpf; F, 48hpf). Oprm1 protein levels measured by IP-WB in control and miR-212 morphant embryos after morphine (10 nM and 10μM) treatment (G and I, 24hpf; H and J, 48hpf). C-: Negative control Mo: morphant. IgG HC: immunoglobulin gamma heavy chain.** Morphine regulates both miR-212 and *oprm1* expression, inducing an upregulation of both molecules at 24 hpf, but a downregulation at 48 hpf. Results are shown as the fold change respect to control (dotted line) at 24 and 48 hpf. Data are expressed as mean ± SEM. Statistical significance; *, p < 0,05; **, p < 0,01; ***, p < 0,001.

The levels of expression of miR-212 in zebrafish embryos were measured by qPCR after the administration of two different doses of morphine (10nM and 10μM) from 5 hpf (Fig 2). We have previously used these two doses to cover a wide variety of pharmacological responses triggered by morphine. As for a biochemical rationale, we have already proven that both doses bind to mu opioid receptor and could exert different responses [24]. However, the concentrations used can be considered neither clinical nor abusive, since we are not able to evaluate behavioral changes related to these conditions in the zebrafish embryos. Our group has also antagonized these doses of morphine with naloxone, obtaining a clear reversal of the opioid effects [20]. At 24 hpf (Fig 2A), an increase in the levels of the miRNA was observed for both concentrations of morphine, although it was higher at 10μM. In contrast, miR-212 was downregulated at 48 hpf. Additionally, we analyzed the implication of mu opioid receptor in miR-212 expression. After knocking-down the mu opioid receptor, the up-regulation induced at 24 hpf by morphine administration was not observed (Fig 2A). However, the decrease in the expression was not reversed by *oprm1* morpholino at 48 hpf (Fig 2B). Also, as shown in the Figs (Fig 2A and 2B), the levels of miR-212 were not modified in the control *oprm1* morphants. In addition, we measured the levels of the pri-miR-212/132 (Fig 2C and 2D) and we observed a correlation between the expression of the pri-miR and miR-212, except for *oprm1* morphants exposed to 10μM morphine in which an upregulation is observed. Furthermore, we analyzed the differences in the results when zebrafish embryos were exposed to morphine from 20 to 24 hpf, instead of 5 hpf. We observed the same results in the levels of miR-212 in both cases, which possibly indicates that the changes observed at 24 hpf are an acute response to morphine exposure. As miR-212 expression is affected by morphine administration, we analyzed if mu opioid receptor was being regulated by this miRNA (Fig 2E and 2F). After morphine treatment, mu opioid receptor was strongly up-regulated at 24 hpf (Fig 2E). In addition, we have observed a decrease in the levels of *oprm1* at 48 hpf after morphine administration (Fig 2F). However, the knock-down of miR-212 induced an increase in the expression of the receptor, much higher than the observed when miR-212 was present at both 24 and 48 hpf.

Finally, to determine whether these changes observed in the mRNA levels were correlating with the protein levels, we performed an immunoprecipitation-western blot (IP-WB, Fig 2G and 2H). The results obtained with this technique correlate with our qPCR experiments (Fig 2I and 2J). However, Oprm1 was downregulated in miR-212 morphants exposed to 10μM morphine at both 24 hpf and 48 hpf (Fig 2I and 2J) and considering that miR-212 forms a cluster with miR-132, we decided to analyze if both miRNAs could regulate *oprm1* translation.

### miR-212/132 cluster regulates *oprm1* mRNA expression binding to its 3’UTR

**Fig 3:**
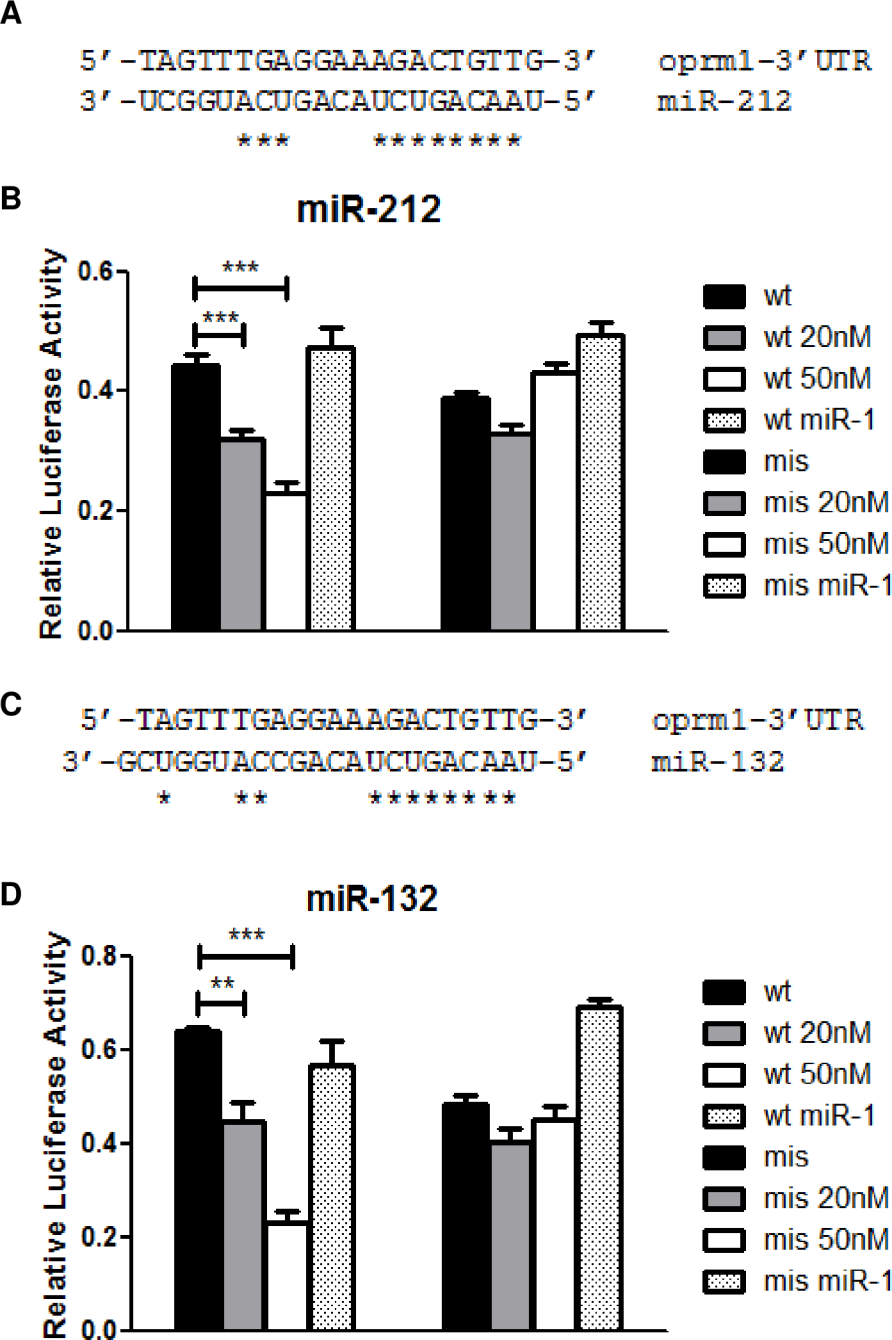
**Complementarity between *oprm1* mRNA 3’UTR and miR-212 (A). and between *oprm1* 3’ UTR and miR-132 (C). Luciferase assay of the binding site for miR-212 in the 3’UTR of *oprm1* (B) and miR-132 (D). wt, unmutated plasmid; mis, mutated plasmid**. Results are shown as the fold change respect to control. The groups transfected with the wild-type plasmid and the plasmid containing the mismatch were cotransfected with 20nM, and 50nM miR-212 or miR-132 mimics, and also with 50nM miR-1 mimics as negative control. The cluster formed between miR-212 and miR-132 regulates *oprm1* expression, as observed in the significative changes when the wild type plasmid is cotransfected with an active form of the miRNAs. Data are expressed as mean ± SEM, N = 8. Statistical significance; ***, p < 0,001.

In order to determine whether these miRNAs were effectively regulating *oprm1* by targeting its mRNA, we performed a bioinformatic analysis of putative binding sites of mu opioid receptor [25]. A possible binding site for miR-212/132 was observed in the 3’ UTR of *oprm1*, as well as an additional site in the second exon of the mRNA. By means of a luciferase assay we confirmed that the binding site in *oprm1* 3’ UTR was actively repressing mu opioid receptor (Fig 3). miRNA mimics (small, chemically modified double-stranded RNAs that mimic endogenous miRNAs by up-regulation of miRNA activity) were cotransfected with the plasmids, and we observed a significant decrease in the luminescence on the unmutated 3’ UTR binding site (wild type, wt). The mutation of the seed region (mismatch, mis) reversed this decrease, indicating that the predicted binding site was physiologically functional. Moreover, the increase on the concentration of miRNA mimics reduced the luciferase activity, but only when cotransfected with the wt plasmid, corroborating our findings. These results prove that miR-212 and miR-132 are binding to the 3’UTR region of *oprm1* mRNA, and physiologically repressing *oprm1* expression (Fig 3). The decrease in the luminescence on both wt and mis plasmids is probably due to the endogenous activity of the miRNAs present in HEK293 cells.

### Morphine modifies miR-212 expression in the midbrain and hindbrain of zebrafish embryos

**Fig 4:**
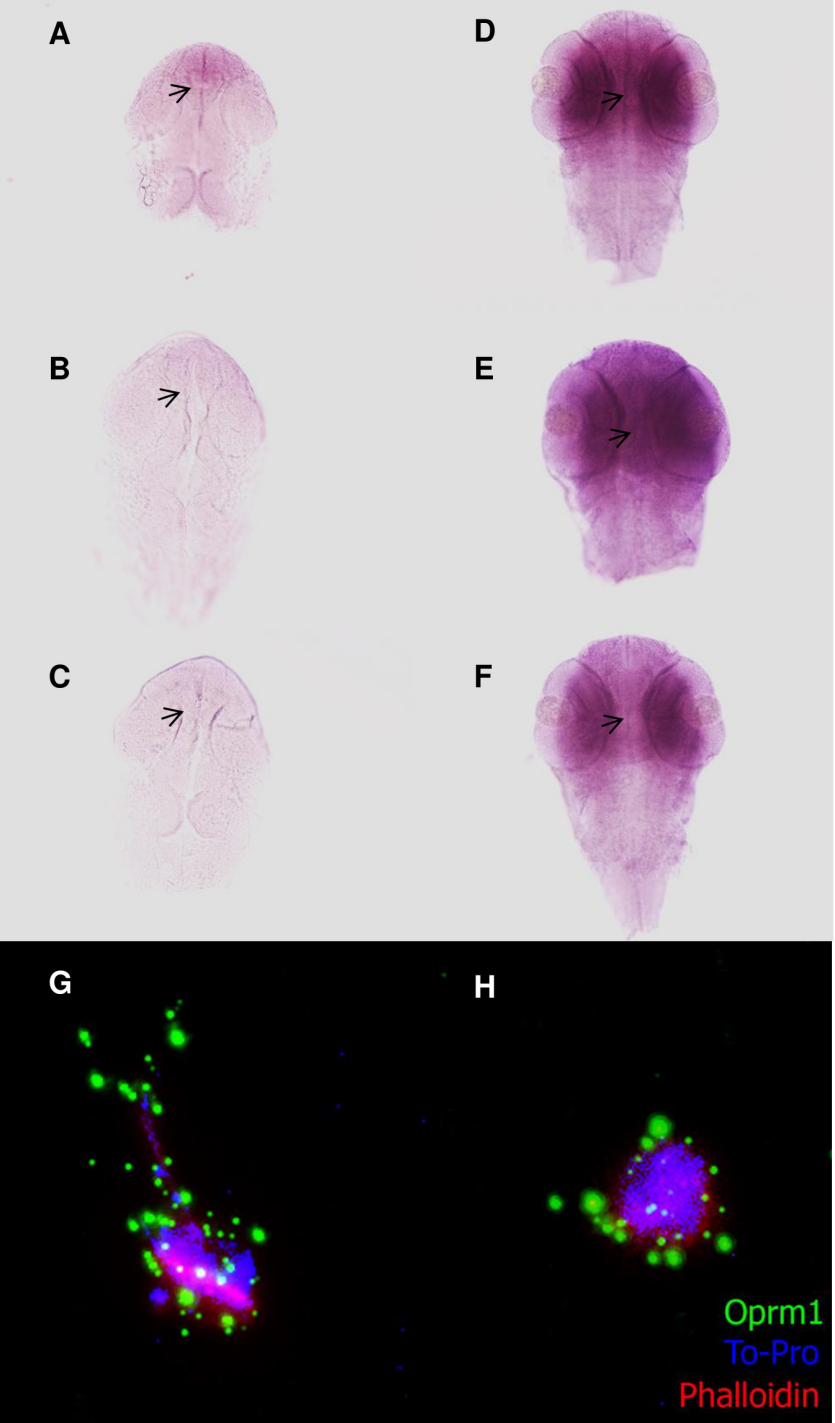
**ISH against miR-212 (A-F) and IHC against Oprm1 (G-H). miR-212 pattern of expression in control (A, D) and after morphine treatment (10 nM (B,E) and 10μM (C,F)) (left, 24hfp; right, 48hpf). Zebrafish primary neuron culture (G, H), control (G) and 1nM morphine (H).** The arrows show the regions in the midbrain and in the hindbrain where the changes observed in miR-212 expression levels are more relevant. The IHC performed on the primary neuron culture showed changes in the levels of Oprm1 (green), which correlates with the results observed in the qPCR and ISH experiments.

The results observed by qPCR were corroborated by using an ISH probe against the pri-miR-212/132 (Fig 4A–4F). These miRNAs are expressed in midbrain and hindbrain, and the expression on these areas correlates with the changes obtained in the qPCR experiments. In addition, we obtained a primary neuron culture and performed an IHC against Oprm1 to determine if the changes observed *in vivo* occurred at the cellular level (Fig 4G and 4H). The expression of the Oprm1 receptor was increased 24 hours after the administration of 1nM morphine (Fig 4H), corroborating the results of the qPCR. The morphology of neurons was also modified, showing a rounded shape after morphine exposure.

### miR-212 expression is regulated by MAPK, calmoduline and PKA

**Fig 5:**
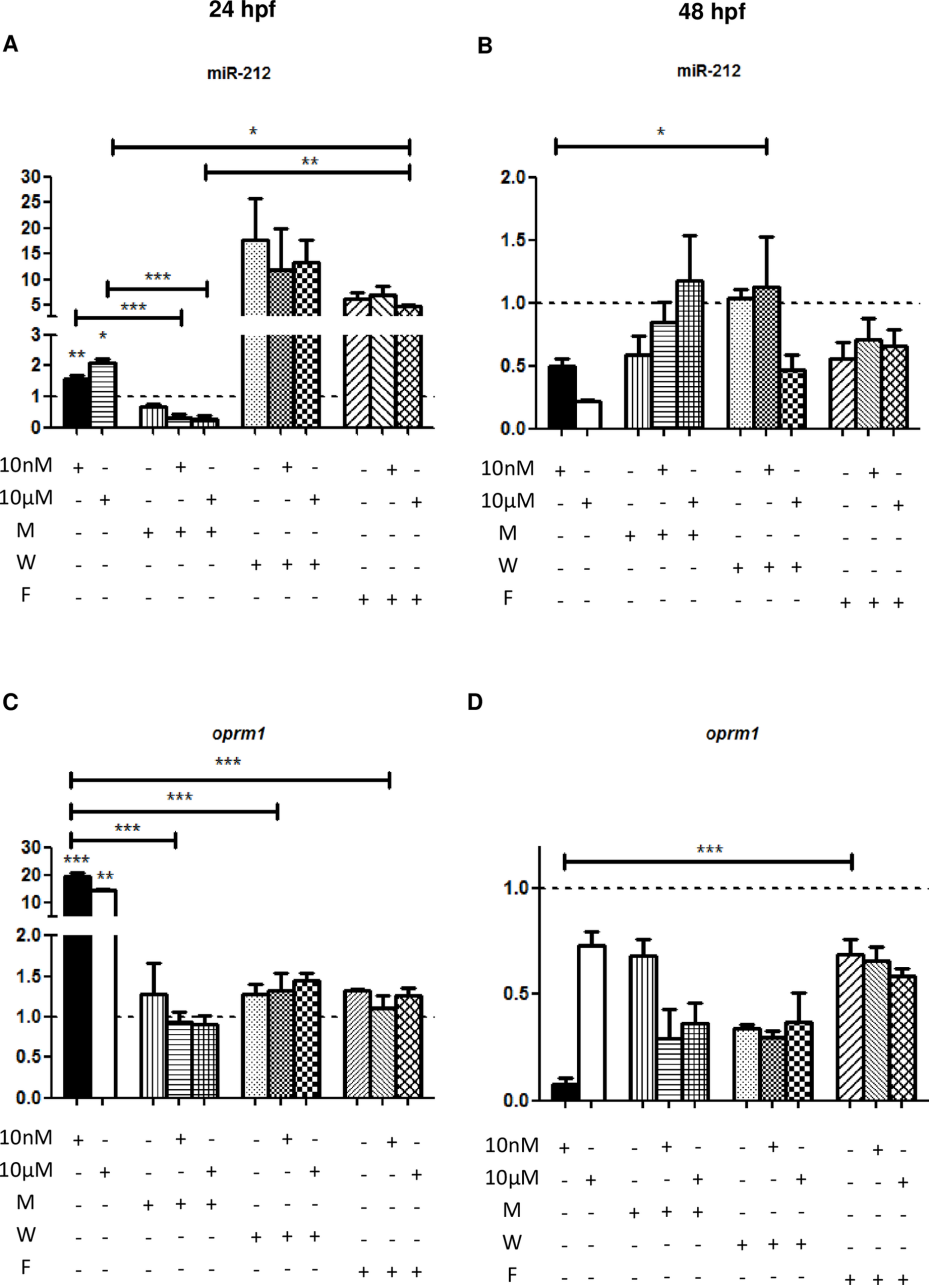
**miR-212 expression levels after morphine administration and inhibition of MEK1/2 (U0126, M), CaM/CaMKII inhibitor (W7, W) and PKA hyperactivation (Forskolin, F) (A, 24hfp; B, 48hpf). *oprm1* expression levels after morphine administration and inhibition of MEK1/2 (U0126, M), CaM/CaMKII inhibitor (W7, W) and PKA hyperactivation (Forskolin, F) (C, 24hfp; D, 48hpf).** Results are shown as the fold change respect to control (dotted line). These results show that the three pathways studied are involved in miR-212 expression, but only MAP kinases are related to *oprm1* changes after morphine administration. Data are expressed as mean ± SEM, N = 3. Statistical significance; *, p < 0,05; **, p < 0,01; ***, p < 0,001.

To analyze in detail the signaling cascade triggered by the activation of Oprm1, the levels of both miR-212 and *oprm1* were studied after the coadministration of morphine and U0126, an inhibitor of MEK1/2 (Fig 5). The upregulation of miR-212 expression after morphine exposure at 24 hpf is reversed to normal levels when the MAP Kinase pathway was blocked (Fig 5A). In addition, we inhibited calmoduline (CaM) and calmoduline kinase II (CaMKII) using W7 to determine the implication of calcium signaling in miR-212 expression. As observed in the Fig 5A, calcium signaling inhibition induces an overexpression of miR-212 in control, 10nM and 10μM groups. Using Forskolin, an activator of PKA, we observed a strong upregulation of this miRNA in the three groups (Fig 5A). In contrast, at 48 hpf, miR-212 levels remained decreased after inhibiting MEK1/2, calcium signaling or activating PKA (Fig 5B). The inhibition of MAP kinase pathway and CaM/CaMKII after 10nM morphine administration did not induce the strong downregulation observed in the 10nM control embryos (Fig 5B). The mild downregulation observed in the 10μM group is potentiated by disrupting MEK and calcium signaling. PKA over-activation mimicked the decrease of 10μM control group, even when coadministrated with morphine. *oprm1* overexpression after morphine exposure is mediated by MEK, and blocked by U0126 at 24 hpf (Fig 5C). Moreover, forskolin prevents the induction of the expression of *oprm1*. At 48 hpf, the downregulation in *oprm1* expression levels is partially reverted after MEK and calcium pathways inhibition and by the increase of cAMP (Fig 5D).

## Discussion

In our analysis of the miR-212/132 cluster, the expression of miR-132 is higher than that observed for miR-212. Furthermore, the sequence of miR-132 is more conserved during evolution when compared to that of miR-212 [26], meaning that the function of this miRNA may be essential during the early stages of development. miR-212 presents the maximum expression at 8 hpf, while the highest expression observed for miR-132 is at 16 hpf. These miRNAs may be related to somite differentiation (directly related to muscle formation) which are being developed at these stages. The importance of miR-132 in muscle differentiation has been previously described by Bijkerk et al. [27]. Several authors have described a downregulation of miRNAs that belong to the same cluster in some diseases [28, 29, 30]. Although the mechanism for this regulation has been studied, it still remains unclear [31]. The downregulation observed in miR-132 levels after knocking-down miR-212 may indicate that miR-212 is regulating the expression of the miRNAs in the cluster, and may have evolutionarily emerged as a regulator for miR-132, which could be crucial during development [32]. The upregulation observed at 24 hpf in the expression of miR-212 after morphine administration is mediated by the activation of the mu opioid receptor, since *oprm1* morphants do not show any significant change. In contrast, a decrease of this miRNA is observed at 48 hpf, but it is not reversed in the morphants. These results indicate that the up-regulation of this miRNA observed after morphine exposure in the early stages of development is mediated by the mu opioid receptor. On the other hand, the decrease of miR-212 levels induced after prolonged morphine administration is mediated by a different pathway than that triggered by the activation of mu opioid receptor, as we have observed in the primiR-212/132 levels after 10μM morphine administration in *oprm1* morphants. Previous results from our group have demonstrated a differential expression of miR-132 and miR-212 in 48 hpf zebrafish embryos [33]. However, the knock down of the mu opioid receptor did not modify the levels of expression of miR-212, thus the physiological expression of this miRNA is not controlled by Oprm1, but this receptor is responsible for the changes observed after morphine exposure. Hence, Oprm1 activation by morphine modifies the levels of miR-212. Moreover, the changes in the levels of its expression were also observed in the pri-miR-212/132 levels. These results suggest that morphine is not only preventing the degradation of the miRNA but effectively inducing its expression. However, the regulation of the expression and maturation of these miRNAs may be mediated by any other factors, leading to a differential expression of these two miRNAs during the development, as observed in *oprm1* morphants exposed to 10μM morphine. Several authors have already observed similar findings as a result of a post-transcriptional regulation of clustered miRNAs [34]. The localization of the precursor, as seen in the ISH pictures, also correlates with the results measured by qPCR. We have also observed several changes in the shape of the neurons in the primary neuron culture, possibly due to an alteration in the differentiation, as previously described by other authors [14, 35].

An upregulation of *oprm1* levels at 24 hpf has also been obtained after the administration of morphine. This receptor has been shown to induce its own expression in a feedforward mechanism after an acute morphine dose in zebrafish embryos [20]. In contrast, a decrease in the levels of *oprm1* mRNA is observed at 48 hpf, probably due to a prolonged activation of the receptor or to a reduction in the expression exerted on its promoter by some of the known repressors, such as MeCP2 [36] or PU.1 [37]. On the other hand, we have observed an increase at the protein levels after morphine administration. These results suggest that the reduction on miR-212 levels observed after the exposition to morphine is preventing the blockage of the translation. Furthermore, the role of the endogenous opioids in *oprm1* expression has been already studied. These molecules, widely expressed in the brain, are affected by morphine and could also be interacting with the normal activation of Oprm1 [38]. After knocking down miR-212, we observed an increase in the expression of *oprm1*. Furthermore, the decrease induced by morphine at 48 hpf was reversed and turned into a mild increase. However, the decrease observed after exposing *oprm1* morphants to 10μM morphine could be mediated by miR-132 overexpression. The differential expression between miR-212 and miR-132 has already been described, and this upregulation could be mediated by other receptor rather than Oprm1. These results show a novel regulation by the cluster formed between miR-212/132 exerted on *oprm1* translation, preventing the translation of *oprm1* mRNA by targeting the binding site on the 3’ UTR. At the same time, this mechanism prevents further overexpression of the levels of *oprm1* after morphine exposure. In summary, these results indicate that morphine, through the activation of Oprm1, induces miR-212 overexpression. This epigenetic factor, along with the other transcriptional regulators, reduces the translation of *oprm1* mRNA, controlling the expression of the receptor at the post-transcriptional level. Furthermore, the regulation could be also mediated by miR-132, and other pathways such as neurotrophins or other opioid receptors.

To study the molecular mechanisms underlying Oprm1 activation, we selectively inhibited several pathways related to opioid activity, such as MAP Kinases, Calmoduline/Calmoduline Kinase II and Protein Kinase A (PKA) (Fig 5). We have demonstrated that the upregulation of miR-212 induced at 24 hpf is mediated by the MAP kinase pathway (through the activation of MEK1/2) using the inhibitor U0126. The overexpression of *oprm1* is also reversed after the addition of U0126. However, we have observed a decrease in the levels of miR-212 and *oprm1* at 48 hpf compared to 24 hpf. This reduction is not fully reverted by U0126, indicating that this response may not only be mediated by the MAP kinase pathway, and hence by the activation of CREB. This transcription factor has been described to induce the expression of miR-212, while the REST protein, binding to RE1 sites, has been demonstrated to reduce the activity of its promoter [39] as well as for *oprm1* [36]. Besides, methyl CpG binding protein 2 (MeCP2) is also involved in the reduction of the expression of this miRNA forming an epigenetic protein complex [15]. Hence, the repression exerted on these two genes is also probably mediated through the activation of MeCP2 by binding to methylated RE1 sites. In addition, our results show that calcium signaling is necessary to induce the upregulation of *oprm1* after morphine administration, since W7 prevented the overexpression of the mRNA.

Furthermore, the induction of the activity of MeCP2 is mediated by calmodulin (CaM) and calmodulin kinase II (CaMKII) [40]. The activation of mu opioid receptor is known to block calcium channels via the alfa subunit Gi (α Gi), modifying the levels of intracellular calcium [41]. CaM induces MAP kinase activation mediated by PKC, RAS and Raf1 activation [42], therefore triggering CREB phosphorylation. In addition, the activation of βγ subunits induces the release of the intracellular deposits of calcium of the RE mediated by inositol triphosphate (IP_3_) through phospholipase C (PLC) activation [43]. In addition, cAMP signaling inhibition has been demonstrated to regulate morphine response [44]. Therefore, while CaM/CaMKII inhibition reduces MeCP2 phosphorylation and activation, it also increases CREB activity and miR-212 expression, as we have shown after using the inhibitor W7. On the other hand, PKA activation mediated by forskolin may be inducing CREB phosphorylation and therefore activating miR-212 and *oprm1* transcription. In fact, the levels of miR-212 are downregulated compared to the control group. This indicates that MAP kinase signaling, activated by other pathways (such as neurotrophins, as described by Numakawa [45] and Remenyi [46] and) is necessary to maintain miR-212 physiological levels. If CaMKII activity is inhibited by W7, we observe a strong increase in the levels of the microRNA. CaMKII phosphorylation of Ser421 of MeCP2 inhibits the expression of this miRNA by blocking transcription through the binding to RE1 sites [15]. The loss of activity in this calcium pathway induces a loss of phosphorylation on MeCP2, and thus reduces its activity. On the other hand, this epigenetic inhibition does not control mu opioid receptor expression, as CaM/CaMKII inhibition does not involve any significant change. βγ subunits activation of PLC and posterior IP_3_ release induces the liberation of intracellular calcium stored in the endoplasmatic reticulum, and the activation of CaMKIV [47]. This kinase induces the activation of MAP kinase pathway [48], and the posterior phosphorylation of CREB, resulting in an overexpression of miR-212 and *oprm1*. In addition, miR-212 targets *oprm1* mRNA blocking the translation mediated by RISC complex [49], preventing further overexpression of the receptor. This mechanism serves as a feedback control of mu opioid receptor expression: morphine induces miR-212 and *oprm1* transcription, increasing their levels. This miRNA, on the other hand, targets *oprm1* mRNA and reduces the levels of the protein.

Furthermore, the changes in the methylation pattern of miR-212 and *oprm1* have been already studied [34, 35]. MeCP2 is actively repressing their promotor binding to RE1 sites and preventing the overexpression observed after morphine administration. Since the inhibition of CaM/CaMKII induces an increase of the expression of these two genes, our results indicate that MeCP2 phosphorylation on Ser421 [40] is necessary for the activity of this epigenetic factor. PKA activation by forskolin partially rescues the normal expression of these molecules. CREB phosphorylation mediated by PKA could provoke an induction of the expression of miR-212 and *oprm1*, but as MeCP2 is still activated and bound to the promoter, the transcription is partially blocked, as has been described in the regulation of other genes of many cellular processes [50].

**Fig 6:**
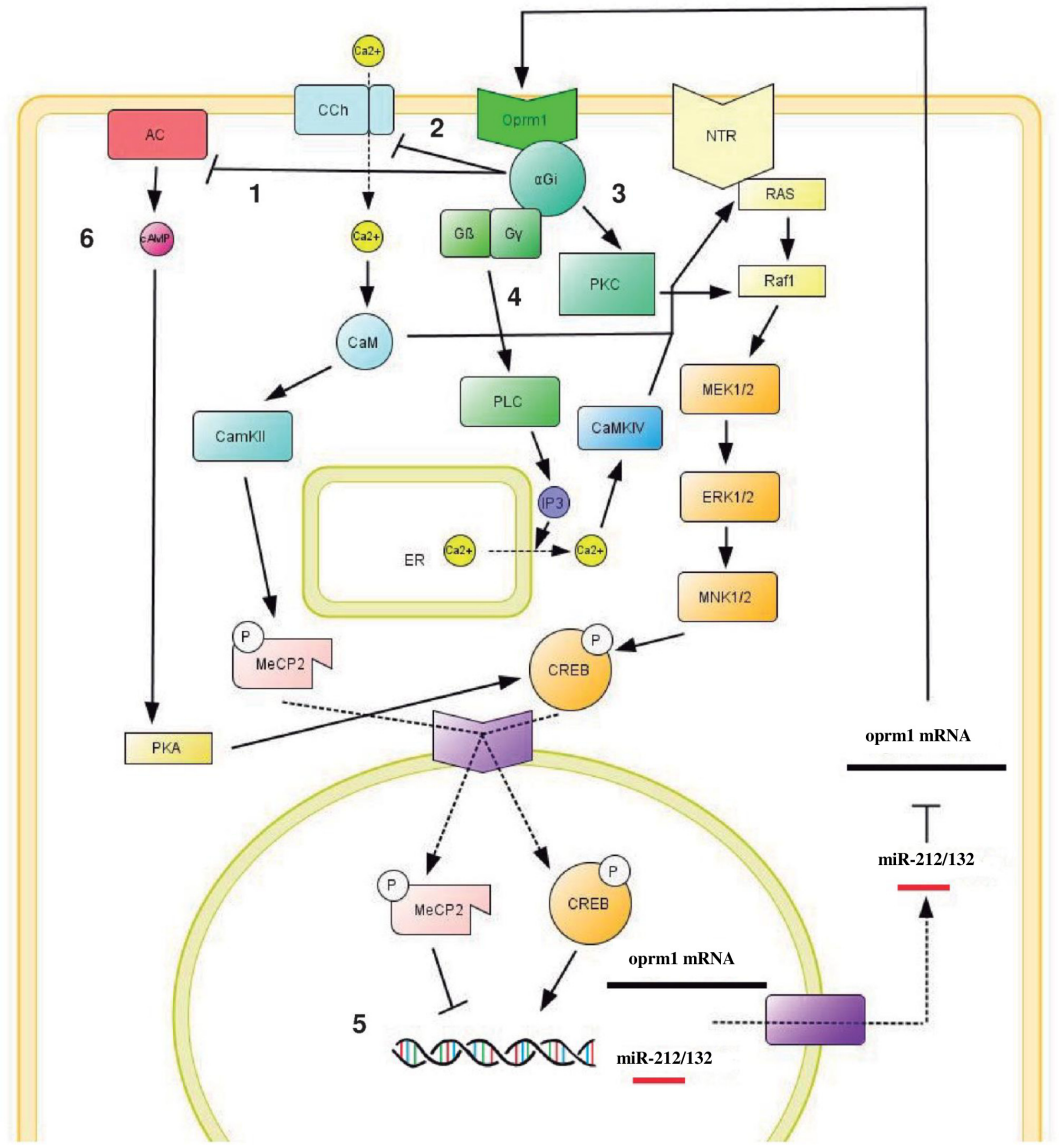
Proposed mechanism for the relationship between the mu opioid receptor (Oprm1), activated by morphine, and the cluster miR-212/132. The responses observed at 24 hpf involve different steps: 1: inhibition of adenylate cyclase mediated by α Gi. 2: α Gi subunit closes calcium channels 3: PKC activation induces MAP kinase pathway, phosphorylating CREB. 4: βγ subunits induce calcium release of endoplasmatic reticulum. The downregulation observed at 48 hpf involves two steps: 5: changes in the methylation patterns on miR-212 and *oprm1* promoters induce MeCP2 repression, blocking transcription, 6: PKA overactivation. CREB phosphorylation induces miR-212 and *oprm1* expression. In addition, miR-212 targets *oprm1* 3’UTR and prevents its translation, controlling the levels of the receptor. At 48 hpf, changes in the methylation pattern and the joining of MeCP2 to the promoters of miR-212/132 and *oprm1* prevents CREB action, downregulating both genes.

It is clear, taking the above into consideration, that there is a relationship between the activity of the mu opioid receptor and miR-212. We propose the following mechanism after morphine administration (Fig 6): the activation of Oprm1 (light green) induces several intracellular changes; **1**: activation of α Gi subunit (dark green) inhibits adenylyl cyclase (AC, red), interrupting the production of cAMP and preventing the activity of Protein Kinase A (PKA, yellow); **2**: In addition, α Gi subunit closes calcium channels (CC, light blue); **3**: Oprm1 activation of Protein Kinase C (PKC, green) induces the activation of RAS and Raf1, which activate MAP Kinase pathway (MEK, ERK and MNK1/2, orange), inducing the phosphorylation of CREB (orange); **4**: βγ subunits induce the release of IP_3_, and the posterior release of intracellular calcium, stored at the endoplasmatic reticulum (ER). In contrast, the downregulation observed in all the experimental groups at 48 hpf seems to be mediated by several factors, but not by Oprm1 activation. Moreover, the expression of miR-212 and *oprm1* is clearly reduced, indicating that the mechanism undergoing after morphine administration must be different. The regulation mechanism involved in the responses observed is mediated by two processes; **5**: the changes on the methylation pattern in miR-212 and *oprm1*, probably in RE1 sites, inducing the repression of the transcription mediated by MeCP2 and **6**: cAMP overproduction after prolonged morphine administration induces PKA activation [39].

These mechanisms present novel insights into the signaling cascade triggered by the activation of Oprm1. These results, together with receptor endocytosis studies, may explain the first steps of morphine activity in the CNS.
